# Microsatellites with Macro-Influence in Ewing Sarcoma

**DOI:** 10.3390/genes3030444

**Published:** 2012-07-23

**Authors:** Michael J. Monument, Kirsten M. Johnson, Allie H. Grossmann, Joshua D. Schiffman, R. Lor Randall, Stephen L. Lessnick

**Affiliations:** 1 Center for Children’s Cancer Research, Huntsman Cancer Institute, University of Utah, Salt Lake City, UT 84112, USA; E-Mails: Michael.Monument@hci.utah.edu (M.J.M.); Kirsten.Johnson@hci.utah.edu (K.M.J.); Joshua.Schiffman@hci.utah.edu (J.D.S.); Lor.Randall@hci.utah.edu (R.L.R.); 2 Sarcoma Services, Department of Orthopedic Surgery, University of Utah, Salt Lake City, UT 84108, USA; 3 Department of Pathology and Program in Molecular Medicine, University of Utah, Salt Lake City, UT 84112, USA; E-Mail: Allie.Grossmann@hsc.utah.edu; 4 Division of Pediatric Hematology/Oncology, University of Utah, Salt Lake City, UT 84108, USA; 5 Department of Oncological Sciences, University of Utah, Salt Lake City, UT 84112, USA

**Keywords:** EWS/FLI, ETS family transcription factors, transcriptional regulation, oncogenesis, microsatellite DNA, microsatellite polymorphisms

## Abstract

Numerous molecular abnormalities contribute to the genetic derangements involved in tumorigenesis. Chromosomal translocations are a frequent source of these derangements, producing unique fusion proteins with novel oncogenic properties. EWS/ETS fusions in Ewing sarcoma are a prime example of this, resulting in potent chimeric oncoproteins with novel biological properties and a unique transcriptional signature essential for oncogenesis. Recent evidence demonstrates that EWS/FLI, the most common EWS/ETS fusion in Ewing sarcoma, upregulates gene expression using a GGAA microsatellite response element dispersed throughout the human genome. These GGAA microsatellites function as enhancer elements, are sites of epigenetic regulation and are necessary for EWS/FLI DNA binding and upregulation of principal oncogenic targets. An increasing number of GGAA motifs appear to substantially enhance EWS/FLI-mediated gene expression, which has compelling biological implications as these GGAA microsatellites are highly polymorphic within and between ethnically distinct populations. Historically regarded as junk DNA, this emerging evidence clearly demonstrates that microsatellite DNA plays an instrumental role in EWS/FLI-mediated transcriptional regulation and oncogenesis in Ewing sarcoma. This unprecedented role of GGAA microsatellite DNA in Ewing sarcoma provides a unique opportunity to expand our mechanistic understanding of how EWS/ETS fusions influence cancer susceptibility, prognosis and transcriptional regulation.

## 1. Introduction

**Figure 1:**
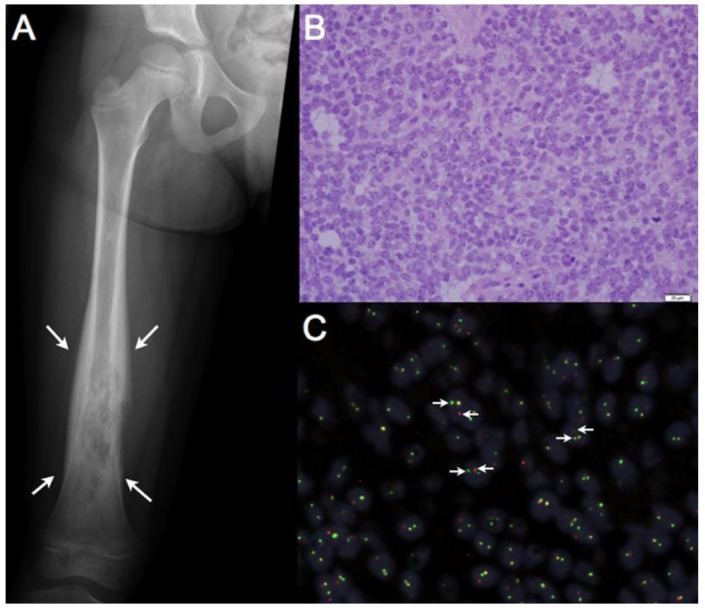
Ewing sarcoma is an aggressive bone associated malignancy characterized by chromosomal translocations. (**a**) Classic radiographic appearance of Ewing sarcoma: an expansile, destructive lesion (outlined by white arrows) of the femoral diaphysis (shaft) in a skeletally immature patient. Ewing sarcomas can also present as an isolated soft tissue mass, although this is less common; (**b**) 400× magnification of a Hematoxylin and Eosin (H & E) stained section from a Ewing sarcoma tumor. Microscopically, these tumors are characterized by sheets of small round cells with a high nuclear-to cytoplasmic ratio; (**c**) Break-apart Fluorescence *in situ* Hybridization (FISH) showing EWSR1 rearrangements in 84% of tumors cells, confirming the diagnosis of Ewing sarcoma. Dual, non-overlapping, 5’-*EWSR1* probes (red) and 3’-*EWSR1* probes (green) detect the presence of a chromosomal rearrangement; when the red and green probes are split into two distinct signals (white arrows) a chromosomal rearrangement is identified, whereas an orange signal indicates an intact *EWSR1* locus.

Aberrant chromosomal translocations are common observations in cancer and in many instances these events give rise to chimeric fusion products with novel biological and cellular functions. Many of these chimeric fusion proteins function as oncogenic transcription factors, essential for cellular transformation and/or critical malignant cellular phenotypes [1,2]. Ewing sarcoma is a highly aggressive bone associated malignancy primarily affecting children and young adults, ubiquitously characterized by and derived from a balanced chromosomal translocation [3,4]. Ewing sarcoma belongs to a larger class of malignancies referred to as *sarcomas*, a term ascribed to a heterogeneous grouping of tumors derived from, or highly associated with connective tissue elements and mesenchymal precursors (Figure 1). Ewing sarcoma is an aggressive malignancy, with significant metastatic potential. Roughly 20% of patients present clinically with detectable metastatic disease, where survival ranges from 60–75% in patients with localized disease and plummets to <20% in those with local recurrence or metastatic disease [3,5]. 

**Figure 2:**
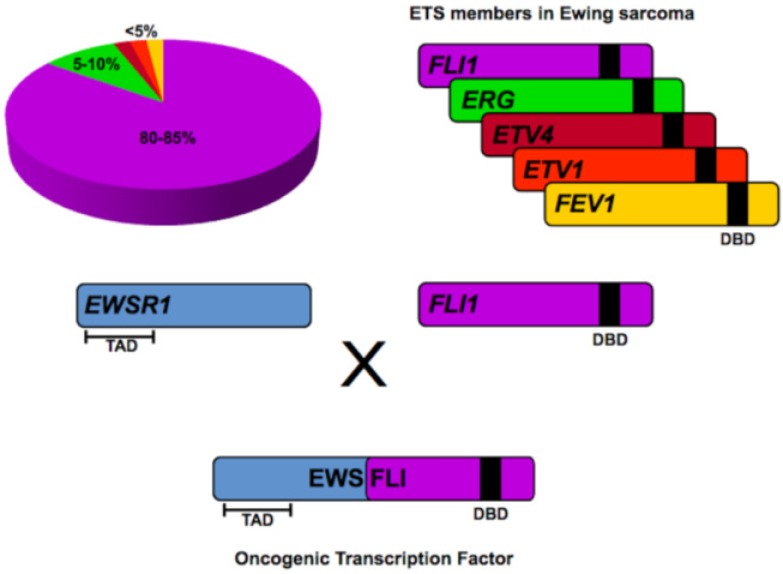
EWS/ETS fusions in Ewing sarcoma. EWS/FLI fusions comprise 80–85% of all translocations in Ewing sarcoma. Translocations involving other ETS family members such as *ERG*, *ETV4*, *ETV1* and *FEV1* are less common. In all instances, the transcriptional activating domain (TAD) in the N-terminus of EWS is fused to the C-terminal DNA binding domain (DBD) of the ETS family member. The resultant chimeric fusion protein functions as a potent oncogenic transcription factor responsible for tumorigenesis in Ewing sarcoma.

Virtually all Ewing sarcoma tumors harbor a somatic translocation, fusing the *EWSR1* gene (encoding the EWS protein) on chromosome 22 with a member of the ETS family of transcription factors, most commonly *FLI1* (encoding the FLI protein), located on chromosome 11 [t(11;22)(q24;q12)]. The EWS/FLI fusion product is observed in 80–85% of cases, with highly related fusions such as EWS/ERG, EWS/FEV, EWS/ETV1 and EWS/ETV4 occurring less frequently (reviewed in Sankar and Lessnick, 2011 [6]). In Ewing sarcoma, chimeric EWS/ETS fusion products function as an aberrant oncogenic transcription factor, mediated by the transcriptional activating amino-terminus of EWS fused in frame to the DNA binding carboxy-terminus of the ETS transcription factor (Figure 2). Numerous studies have since confirmed that malignant transformation in Ewing sarcoma is dependent on EWS/ETS fusions and consequently, these chimeric oncoproteins are regarded as critical upstream regulators of the transcriptional hierarchy in this cancer [7,8,9]. The prevailing influence of EWS/FLI in Ewing sarcoma provides a unique opportunity to further characterize the oncogenic properties of EWS/ETS proteins, with hope that this growing body of knowledge will allow for a greater understanding of the molecular basis of oncogenesis and facilitate the development of more targeted, clinically efficacious therapy for this devastating malignancy.

## 2. ETS Family of Transcription Factors

The ETS (E-twenty-six) transcription factors belong to a family of highly evolutionarily conserved DNA binding proteins instrumental for a variety of critical cellular processes including proliferation, cellular differentiation, angiogenesis, lymphoid cell development, apoptosis and cell migration (reviewed in ref [10]). Given these important functions, it is of no surprise that dysregulation of numerous ETS family members is commonly observed in cancer. For example, in 50–70% of prostate cancers, chromosomal rearrangements involving ETS-members have been observed [11,12]. In many instances, these rearrangements position the androgen-receptor regulatory element, *TMPRSS2*, directly upstream of the ETS-member, *ERG,* resulting in a hormone-driven overexpression of this transcription factor in prostate cells [11]. In contrast, as this review will expand upon, fusion of the ETS-DNA binding to the transcriptional activating domain of EWS in Ewing sarcoma results in a transcription factor with unique biological properties responsible for oncogenic transformation [7,13,14]. 

Twenty-eight distinct ETS-family members have been identified in humans, which are further categorized into four ETS-subfamilies of more highly related members [15,16]. Common to all ETS-family members is a highly conserved DNA binding domain referred to as the ‘*ETS domain.*’ Structurally, this ‘*ETS domain*’ is a winged helix-turn-helix DNA binding domain composed of about 85 amino acids [17]. This highly conserved DNA binding domain permits binding of ETS-family members to an invariable GGAA/T core DNA target, flanked by nucleotides which facilitate specific ETS-member targeting and cooperative protein-protein interactions [16,18,19]. Two general categories of ETS binding sites have been characterized, which include a high-affinity ETS consensus site located 20–40 bp upstream of the transcriptional start site and a lower affinity consensus site further upstream in the promoter/enhancer element [15,16]. The high-affinity ETS consensus sites afford redundant ETS-member occupancy and gene regulation, are protected from DNA methylation and are associated with basal housekeeping genes. In comparison, the low-affinity sites are modified by simple flanking base substitutions, are frequently adjacent to binding sites for other cooperative transcription factors and are felt to provide a mechanism where individual ETS-members can regulate a distinct cell or tissue-specific transcriptional signature [15,16,20].

## 3. EWS/FLI in Ewing Sarcoma

EWS/FLI and EWS/ERG fusions compromise 80–85% and 5–10% of translocations observed in Ewing sarcoma, respectively [6,13]. Wild-type FLI and ERG are closely related proteins grouped within the ETS class I subfamily. As with other ETS-members, they bind DNA with preference for the traditional ETS high-affinity consensus sequence (ACCGGAAGT) via the highly conserved C-terminal ‘ETS domain’ and possess a weak N-terminus transcriptional activating domain [7,21]. Both function as important regulators of hematopoiesis, B-cell development and vasculogenesis [22,23,24]. Given the predominance of EWS/FLI fusions in Ewing sarcoma, the biology of wild type and fusion-associated FLI has been most thoroughly characterized. In contrast, the precise biology of wild type EWS remains ill-defined, however reports indicate wild type EWS functions as an RNA binding protein and participates in alternative RNA splicing [25,26,27]. 

Functional investigations over the last two decades clearly demonstrate that the biological properties of the EWS/FLI chimera are vastly distinct from wild-type FLI. For instance, while both FLI and EWS/FLI share affinity for the ETS consensus site, the EWS/FLI chimera is a substantially more potent transcriptional activator than wild-type FLI [7,14]. Additionally, ectopic expression of EWS/FLI in NIH 3T3 fibroblasts induces oncogenic transformation whereas wild-type FLI does not [14]. Silencing of EWS/FLI expression in patient-derived Ewing sarcoma cell lines reverses the oncogenic phenotype [8,28]. Interestingly, wild-type FLI is not expressed in Ewing sarcoma cells [8]. Furthermore, the transcriptional signature and genomic targeting of EWS/FLI in Ewing sarcoma is markedly different from wild-type FLI [29], despite a shared affinity for ETS consensus sites [14,30]. 

## 4. EWS/FLI Fusions Mediate Gene Dysregulation via a GGAA Microsatellite Response Element

Genome-wide microarrays have identified >1000 EWS/FLI-regulated genes, including indirect and direct gene targets [8,28,31]. Interestingly, ~80% of these are down-regulated targets. Subsequent chromatin immunoprecipitation approaches, including ChIP-chip and ChIP-seq have further characterized many direct EWS/FLI targets [29,32,33]. Many of the identified up- and down-regulated targets are associated with oncogenic processes described in a variety of other cancer models. However, the most highly regulated and bound target observed across multiple data sets is the gene *NR0B1* (also called *DAX1*) [8,28,33]. NR0B1 is an orphan nuclear receptor, a member of the sex-steroid receptor family, and is important for development of the hypothalamus-pituitary-adrenal-gonadal axis and sex determination [34,35]. NR0B1 has no prior associated role in oncogenesis, which is compelling given the results of the aforementioned microarray and ChIP-chip datasets. Interestingly, *NR0B1* is not bound or transcriptionally regulated by wild-type FLI [29,36]. Numerous independent reports have further validated that *NR0B1* is upregulated, a direct EWS/FLI target, and highly expressed in Ewing sarcoma. Additional functional assessments have shown that in patient-derived Ewing sarcoma cell lines, dysregulated *NR0B1* expression is necessary for oncogenic transformation [28,32,33,36,37]. 

Genome-wide localization studies have established that EWS/FLI highly occupies the *NR0B1* promoter. Mutational experiments have further demonstrated that a 500 bp region, roughly −1.6 kb upstream from the *NR0B1* transcriptional start site is required for EWS/FLI-mediated DNA binding and gene activation [32]. Within this 500 bp region is a 102 bp microsatellite characterized by a series of repetitive GGAA tetra-nucleotide repeats. Numerous investigations have demonstrated that EWS/FLI-mediated binding and activation of *NR0B1* is dependent on this repetitive element [32,33,37]. Interestingly, the highly enriched *NR0B1* promoter does not contain the traditional high-affinity ETS consensus site (ACCGGAAGT) [32,33]. Luciferase reporter constructs and electrophoretic mobility shift assays (EMSA) have further validated the *in vitro* specificity and affinity of EWS/FLI for both the 102 bp *NR0B1* GGAA microsatellite and similar synthetic GGAA microsatellite constructs [30,32]. This data provides compelling evidence that the GGAA microsatellite of the *NR0B1* promoter functions as an “*EWS/FLI response element*,” necessary for DNA binding and gene activation. Of the twenty-eight distinct ETS-members in humans, only 5 have been observed in chromosomal rearrangements with *EWS* in Ewing sarcoma (EWS/FLI, EWS/ERG, EWS/FEV, EWS/ETV1 and EWS/ETV4). All of these related fusion proteins are capable of binding the 102 bp *NR0B1* GGAA microsatellite and activate gene expression [30,32]. Wild-type ETS-members can also bind the GGAA microsatellite; however, unlike EWS/ETS fusions, binding to these elements does not activate gene expression [30].

## 5. Microsatellite Constitution Influences EWS/FLI Binding and Gene Activation

**Figure 3:**
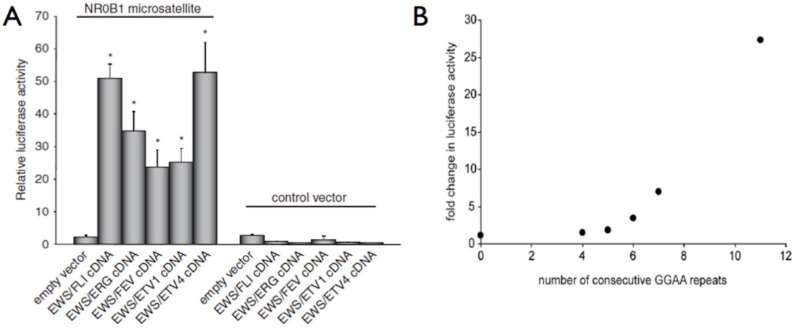
EWS/ETS fusion proteins bind DNA and regulate gene expression via a GGAA microsatellite response element. (**a**) In luciferase reporter constructs, all five EWS/ETS fusions can activate gene expression via the 102 bp *NR0B1* microsatellite; (**b**) Using similar reporter constructs, an increasing number of GGAA motifs, beyond a threshold of four, results in increased gene expression. Panel A reproduced with permission from *Gangwal et al*., *Genes Cancer*. *2010 February 1*; *1*(*2*):*177–187* [30].

ETS family members are commonly known to bind DNA in a monomeric configuration with a characteristic DNAase I footprint of 14–15 bp, although only 9–10 bp are required for sequence specificity [38]. At the aforementioned high-affinity DNA sites, ETS-members bind as monomers, whereas at the lower-affinity, divergent DNA sites, ETS-members often bind as heterodimers in a cooperative fashion with other cell/lineage specific transcription factors [16]. In Ewing sarcoma, EWS/FLI appears to bind to GGAA microsatellites as a homodimer and requires a minimum of 4 consecutive GGAA motifs (16 bp) for binding and gene activation [30,32]. Importantly, beyond a threshold of 4–6 repeats, an increasing number of GGAA motifs results in a proportional increase in EWS/FLI-mediated gene expression in both synthetic reporter constructs and *bona fide* targets, such as *NR0B1* (Figure 3) [29,30,32,33,36,37]. Genome-wide localization data further supports these observations, as sites of EWS/FLI enrichment are greatest in regions with microsatellite elements containing 12–14 consecutive GGAA motifs [29,33]. 

**Figure 4:**
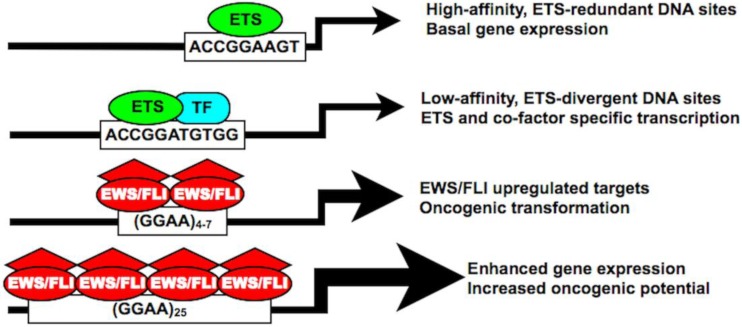
The EWS/FLI chimera possesses unique DNA binding affinities and biological properties distinct from native ETS family members. Both high- and low-affinity ETS DNA binding sites are characterized by a core ACCGGAA/T consensus sequence facilitating both ETS-redundant and ETS-divergent transcriptional regulation. In Ewing sarcoma, EWS/FLI also binds the traditional ETS-consensus sequence, but shows increased preferencefor a GGAA-containing microsatellite. In certain upregulated targets, this GGAA microsatellite response element is required for DNA binding and gene activation, which proportionately increases with an increasing number of GGAA motifs. “TF” = transcription factor.

Collectively, these findings demonstrate an unprecedented role for microsatellite elements as direct EWS/FLI-transcriptional response elements in Ewing sarcoma. Because an increasing number of GGAA motifs substantially augments target gene expression, it is possible that the EWS/FLI chimeric protein has an increased affinity for larger microsatellites. Alternatively, larger microsatellites may facilitate the recruitment of additional EWS/FLI homodimers to produce a synergistic effect on transcriptional activation. Further studies are needed to evaluate these potential mechanisms (Figure 4).

## 6. GGAA Microsatellites Identify Other Potential EWS/FLI Targets and Epigenetically Regulated Enhancer Loci

The compelling evidence linking EWS/FLI-mediated transcriptional regulation of *NR0B1* in Ewing sarcoma to a GGAA microsatellite response element prompted the hypothesis that additional GGAA microsatellite containing genes may be critical targets for oncogenic transformation or other cancer-related phenotypes. By comparing EWS/FLI transcriptional microarray data-sets with genome-wide EWS/FLI localization data, numerous microsatellite-containing direct EWS/FLI targets have been identified [29,32,33,39]. For examples, in ChIP-chip experiments, a promoter microarray was used to assess ~17000 promoters spanning −5.5. kb to 2.5 kb relative to the transcriptional start site, which identified ~900 direct targets. Of the top 134 EWS/FLI-bound genes, a GGAA microsatellite was identified in the promoter region of 12 genes [32]. As previously mentioned, the *NR0B1* promoter was the most highly enriched region, while the remaining GGAA microsatellite-containing genes were dispersed throughout the top 134 bound targets in no particular rank distribution. *Caveolin-1* (*CAV1*) was another GGAA microsatellite containing EWS/FLI target and encodes a critical membrane-associated protein involved in clathrin-independent endocytosis [40]. Dysregulation of *CAV1* has been associated with the metastases in other cancer models [41] and expression of CAV1 is necessary for maintenance of oncogenic transformation in patient-derived Ewing sarcoma cell lines [42]. Using a comprehensive computational mapping of the human genome screening for GGAA microsatellites, another GGAA microsatellite-containing, upregulated target, *GSTM4*, was identified. GSTM4 belongs to a family of glutathione detoxifying enzymes and in patient-derived Ewing sarcoma cell lines, GSTM4 expression is necessary for maintenance of oncogenic transformation [39]. Overexpression of this protein also increases chemoresistance to a chemotherapeutic agent commonly used in Ewing sarcoma, etoposide [39]. Additionally, in a small clinical series, increased expression of GSTM4 in primary Ewing tumors was associated with a lower overall survival [39]. Other microsatellite-containing, direct EWS/FLI targets such as *CACNB2*, *FEZV1*, *FCGRT*, *FVT1/KDSR*, *ABHD6* and *KIAA1797* have also been identified, although the functional importance of these targets in Ewing sarcoma has not been determined [32,33].

In the ChIP-seq data-set reported by Guillon *et al*. [33] a total of 246 EWS/FLI occupied regions were identified, 104 of which were characterized by a GGAA microsatellite. The vast majority of EWS/FLI occupancy was localized to intergenic regions (59%), with less frequent occupancy within gene introns, exons and promoter elements. Utilizing published transcriptional microarray data-sets, it was determined that 60% of EWS/FLI-specific binding was located within 2 Mb upstream of the transcriptional start sites of upregulated EWS/FLI targets. Additionally, the distance of the GGAA microsatellite from the transcriptional start site did not correlate with the rank order of gene upregulation in these transcriptional microarrays. Instead, as predicted from numerous *in vitro* assays, the number of GGAA motifs within the microsatellite had a greater influence on EWS/FLI occupancy and gene expression, which was most pronounced at genomic sites with >9 GGAA motifs [33]. In a more recent genome-wide localization study by Patel *et al*. [29] a combination of ChIP-seq and formaldehyde-assisted isolation of regulatory elements (FAIRE) produced a detailed mapping of EWS/FLI enrichment sites: 40% of EWS/FLI binding sites contained a GGAA microsatellite, >60% of these microsatellite elements were located within intergenic regions and global EWS/FLI-enrichment favored microsatellite elements containing 8–14 consecutive GGAA motifs. Greatest enrichment was localized to a region containing a total of 25 GGAA motifs, which corresponded to the *NR0B1* promoter. A fascinating observation from this data-set was that EWS/FLI modifies the local chromatin structure at these GGAA microsatellites, characterized by a nucleosome-deplete enhancer-like signature. Silencing of EWS/FLI rapidly restored nucleosome occupancy and a closed chromatin configuration at these GGAA microsatellites [29]. 

Collectively, these experiments demonstrate three important mechanistic functions of GGAA microsatellites in Ewing sarcoma: first, as response elements instrumental for direct EWS/FLI-mediated transcriptional regulation of important oncogenic targets such as *NR0B1*, *CAV1* and *GSTM4*; secondly, the spatial relationship of these GGAA microsatellites to upregulated targets strongly suggests these elements possess an enhancer-like function; and finally, these microsatellite elements are regions of EWS/FLI-mediated chromatin modification, facilitating a unique transcriptional signature in Ewing sarcoma.

## 7. The *NR0B1* Microsatellite: A Functional Assessment Tool in Ewing Sarcoma Research

The affinity of EWS/FLI for the *NR0B1* GGAA microsatellite and subsequent gene activation mediated by this interaction is well established [29,30,32,33,36,37]. Consequently, the *NR0B1* GGAA microsatellite response element has become a useful molecular tool in Ewing sarcoma research. Since EWS/FLI is regarded as the principal upstream oncogenic transcription factor in Ewing sarcoma, it is a desirable target for drug development. High-throughput drug and small peptide library screening protocols are effective strategies to simultaneously assess large numbers (10,000–50,000) of therapeutic agents potentially active against EWS/FLI. Reporter constructs using the *NR0B1* promoter are now routinely used as a sensitive measure of EWS/FLI inhibition and have assisted in the identification and a more detailed assessment of new drugs and small peptide inhibitors [43,44,45]. Since the precise cell of origin in Ewing sarcoma remains obscure (reviewed in ref [46]), forced expression or repression of EWS/FLI in patient-derived Ewing sarcoma cell lines and other heterologous systems is commonly employed to assess various cellular pathways of perceived importance in transformation and malignant phenotypes. The *NR0B1* promoter provides an ideal positive control for various systems of inducible EWS/FLI expression (unpublished data).

## 8. Microsatellite DNA in Cancer Pathogenesis

Microsatellite DNA constitutes roughly 3% of the human genome, mostly in non-coding regions [47]. Traditionally, these repetitive elements have been regarded as “junk DNA,” with an undetermined genetic function. Microsatellite DNA has been previously investigated as a potential marker of cancer susceptibility, genomic instability, and prognosis. However, the direct influence of GGAA microsatellite response elements on EWS/FLI-mediated transcriptional regulation of critical targets genes defines a completely novel role of microsatellite DNA in oncogenesis. 

Microsatellite instability (MSI) refers to a change in repeat length of microsatellite DNA, typically due to loss of heterozygosity in genes coding for the DNA mismatch repair (MMR) system. In hereditary non-polyposis colorectal carcinomas (HNPCC) and sporadic colorectal carcinomas, inherited or acquired alterations of the DNA mismatch repair system give rise to a mutator phenotype characterized by length expansions or contractions of multiple mono- and di-nucleotide microsatellites, respectively [48,49,50]. MSI-positive colorectal tumors possess defined biological attributes, such as a more common location in the proximal colon, increased patient survival and favorable patterns of chemosensitivity [49,50,51]. Detection of MSI and defects in the DNA mismatch repair system in colorectal cancer has become instrumental for the diagnosis of HNPCC, whereas in sporadic colorectal carcinomas, MSI provides an important prognostic molecular marker [52,53]. However, instability of these microsatellite sequences is more a manifestation of cancer-related genomic instability and these genetic elements do not appear to mediate specific oncogenic transcriptional signatures. Microsatellite instability has also been assessed in Ewing sarcoma, although with discordant findings [54,55,56]. Since it is now known that the number of GGAA motifs clearly influences EWS/FLI-mediated gene expression in Ewing sarcoma, the determination of MSI in these EWS/FLI microsatellite response elements warrants renewed assessment. 

In addition to MSI, microsatellite polymorphisms associated with various genetic loci have also been associated to cancer susceptibility and pathogenesis. In breast cancer for example, overexpression of the epidermal growth factor receptor, EGFR, is a common finding in invasive ductal carcinomas, where EGFR-positive tumors represent an adverse prognostic marker [57,58]. A dinucleotide CA-microsatellite within intron 1 of EGFR has been identified and length-polymorphisms of this microsatellite have been shown to correlate with basal transcription levels of EGFR [59]; however, a direct mechanistic understanding of this association remains unclear. In prostate cancer, a CAG tri-nucleotide has been identified in the first exon of the androgen receptor gene, coding for a polyglutamine tract in the translated protein. An increasing number of CAG motifs has been shown to reduce the transcriptional activity of the androgen receptor [60]. Polymorphisms of this polyglutamine tract in the androgen receptor also appear to be predictive of cancer susceptibility and prognosis: androgen receptors with a CAG microsatellite of ≤16 CAG motifs are associated with a lower disease incidence and less aggressive tumor biology in those with the disease [61,62]. One of the most common tumor suppressors, p53 has been shown to regulate the transcriptional regulation of one of its targets, *PIG3* using a microsatellite response element. However to date, no functional role for PIG3 has been defined in tumorigenesis [63,64].

## 9. Polymorphic EWS/FLI GGAA Microsatellites: A Novel Approach to Ethnic Patterns of Ewing Sarcoma Susceptibility and Prognosis

At present, compared to many other cancer models, the genetic and environmental risk factors for the development of Ewing sarcoma remain obscure [65]. For unknown reasons, considerable ethnic variation exists in the incidence of Ewing sarcoma: the incidence of Ewing sarcoma is greatest in European populations, which is 10- and 2-fold greater than populations of African and Asian descent, respectively [66,67]. This discrepancy is independent of geographic location, suggesting a strong genetic influence for these observations [66]. Additionally, a recent database of >1,700 patients with Ewing sarcoma demonstrated lower overall survival rates in African and Asian populations [68]. To date, no studies have conclusively explained these epidemiological patterns [65,69,70].

By virtue of the repetitive constitution of microsatellite DNA and the predilection of these repetitive elements for non-coding locations, mutational events have rendered microsatellite DNA highly polymorphic in the human population [47,71]. Microsatellite polymorphisms are routinely used in the assessment of heredity, and phylogenetic mapping of ethnically distinct human populations [72]. Given the mechanistic importance of GGAA microsatellites in EWS/FLI-mediated gene regulation, we hypothesized that polymorphic GGAA microsatellites within and between ethnically distinct human populations may exist, providing a potential explanation for the aforementioned patterns of Ewing sarcoma susceptibility and prognosis. The GGAA microsatellites of the *NR0B1* and *CAV1* promoters were sequenced from 100 unaffected subjects of European and African descent. Our initial hypothesis favored larger GGAA microsatellites in Europeans given the disproportionately high incidence of Ewing sarcoma in this population.

Results from this study demonstrated that the *NR0B1* and *CAV1* GGAA microsatellites were highly polymorphic in both European and African populations. The *NR0B1* microsatellite was substantially more polymorphic than *CAV1* in both populations, where the number of GGAA motifs ranged from 16–60 and 14–72 in Europeans and Africans, respectively. Additionally, while the characteristics of the *CAV1* promoter microsatellites were similar across both populations, the *NR0B1* microsatellite in African subjects was significantly larger, harboring more repeat motifs, a greater number of repeat segments, and longer consecutive repeats, than in European subjects. The vast majority (>85%) of European *NR0B1* microsatellites were tightly clustered around smaller repeats ranging from 16–26 GGAA motifs, whereas 40% of African microsatellites were characterized by large, multi-segment repeats ranging from 30–72 GGAA motifs [73]. These results were opposite to our original hypothesis, but considering the transcriptional implications of an increasing number of GGAA motifs in these EWS/FLI response elements, these results provoke several biologically intriguing hypotheses: It is possible that the massive *NR0B1* microsatellites commonly observed in Africans do not permit a stoichiometrically favorable environment for EWS/FLI binding and are therefore protective of EWS/FLI-mediated *NR0B1* gene activation. Alternatively, these large repeats may facilitate a toxic level of NR0B1 expression and permit premature cellular termination in the presence of EWS/FLI. It is also possible that the increased number of GGAA motifs observed in Africans has no influence on Ewing sarcoma susceptibility but instead supports an enhanced oncogenic potential of affected cells, contributing to the lower survival rates observed in African populations.

Polymorphisms of the *NR0B1* GGAA microsatellite have been observed across the various Ewing sarcoma cell lines, ranging from 16–26 motifs, which approximates the distribution repeats observed in Europeans. *NR0B1* mRNA levels in the various cell lines is tightly correlated with the number of GGAA motifs [37]. Based on this information, it is possible that EWS/FLI has preference for a narrow range of GGAA repeats in the *NR0B1* microsatellite, a so-called “sweet spot” with a GGAA-configuration conducive to maximal EWS/FLI-mediated gene up-regulation. Given the highly polymorphic nature of the *NR0B1* microsatellite within and across ethnically distinct populations, functional assessment of these massive repeats is needed. Correlating polymorphisms of the *NR0B1* GGAA microsatellite in tumor samples with clinical parameters such as overall survival, metastatic burden, anatomic location and chemosensitivity may provide valuable information and lend to the development of GGAA microsatellite polymorphisms as prognostic biomarkers in Ewing sarcoma. 

## 10. Conclusions

Chromosomal translocations are common molecular events in cancer, often producing novel fusion proteins with oncogenic properties. EWS/ETS chimeras in Ewing sarcoma are prototypical fusion products with unique DNA binding and regulatory properties responsible for tumorigenesis. A fascinating emergent property of EWS/ETS chimeras is their ability to directly modulate gene expression and the local chromatin environment via a tetra-nucleotide, GGAA microsatellite. This not only highlights how chimerism vastly alters the biological attributes of involved ETS-members, but also brings to attention a completely unappreciated role of microsatellite DNA in oncogenic transcriptional regulation. GGAA microsatellites have enabled the identification of novel target genes and have become important molecular tools in Ewing sarcoma research. These GGAA microsatellites are also highly polymorphic in human populations, and given that EWS/ETS-mediated gene expression is highly dependent on the length of these repetitive elements, GGAA microsatellite polymorphisms may also provide a unique opportunity to improve our mechanistic understanding of disease susceptibility and prognosis in Ewing sarcoma. Certainly, in Ewing sarcoma, elements once regarded as “genomic junk,” are proving to play a fundamental role in EWS/ETS-mediated oncogenesis.
